# Postpartum Expulsion of an Infected Intramural Uterine Myoma: Rapid Growth in Pregnancy and Definitive Management by Abdominal Hysterectomy

**DOI:** 10.7759/cureus.92246

**Published:** 2025-09-13

**Authors:** Hitomi Nambu, Aya Shirafuji, Hideaki Tsuyoshi, Makoto Orisaka, Yoshio Yoshida

**Affiliations:** 1 Obstetrics and Gynecology, University of Fukui, Fukui, JPN

**Keywords:** gonadotropin-releasing hormone antagonist, intramural myoma, myoma expulsion, postpartum infection, pyomyoma

## Abstract

Uterine myomas during pregnancy can cause significant complications, but postpartum expulsion is rare, particularly from intramural lesions without a stalk. We report the case of a 40-year-old primigravida whose posterior wall intramural myoma enlarged from 3 cm to 21 cm during infertility treatment and pregnancy. The myoma caused inferior vena cava compression, leading to deep vein thrombosis, and remained stable in size after mid-gestation. Vaginal delivery at term was uneventful. Postpartum, the patient developed recurrent genital bleeding and inflammatory signs. Magnetic resonance imaging initially suggested red degeneration, but later revealed penetration of the myoma into the cervical canal, indicating expulsion. Intraoperative findings showed a necrotic, malodorous myoma; cultures grew *Escherichia coli *and *Morganella morganii*. Histopathology confirmed pyomyoma with extensive neutrophil infiltration. Given the absence of a stalk and the high risk of sepsis and hemorrhage, a total abdominal hysterectomy was performed, resulting in full recovery. This is the first documented case of postpartum intramural myoma expulsion requiring abdominal hysterectomy following gonadotropin-releasing hormone (GnRH) antagonist administration. Early recognition of infection-related degeneration, careful assessment of myoma morphology, and timely surgical intervention are essential to prevent life-threatening complications in similar cases.

## Introduction

Pregnancy complicated by uterine myomas is associated with an elevated risk of perinatal complications, including preterm birth, placenta previa, and placental abruption [1]. Uterine myomas may increase in size during pregnancy. Even when their size before conception appears acceptable, rapid enlargement during gestation can unexpectedly precipitate complications, posing significant challenges in pregnancy management. A less common complication is pyomyoma, a condition characterized by infection and abscess formation in degenerated and necrotic uterine myomas during the postpartum period. This condition can result in severe sepsis, underscoring the importance of early diagnosis and appropriate therapeutic intervention [2]. Additionally, pyomyoma in the postpartum period can weaken the adjacent tissues due to infection, allowing even an intramural myoma to penetrate the myometrium and endometrium, resulting in myoma expulsion. Once expulsion occurs, communication with the external environment is established, which can further exacerbate the infection and lead to increased uterine bleeding [3].

In this report, we present successful management for a case of intramural myoma that exhibited rapid growth during infertility treatment and pregnancy and subsequently manifested as myoma expulsion induced by intramyomatous infection in the postpartum period.

This article was previously presented as an oral presentation at the 52nd Annual Meeting of the Hokuriku Obstetrics and Gynecology Society, held on June 8-9, 2024.

## Case presentation

**Figure 1:**
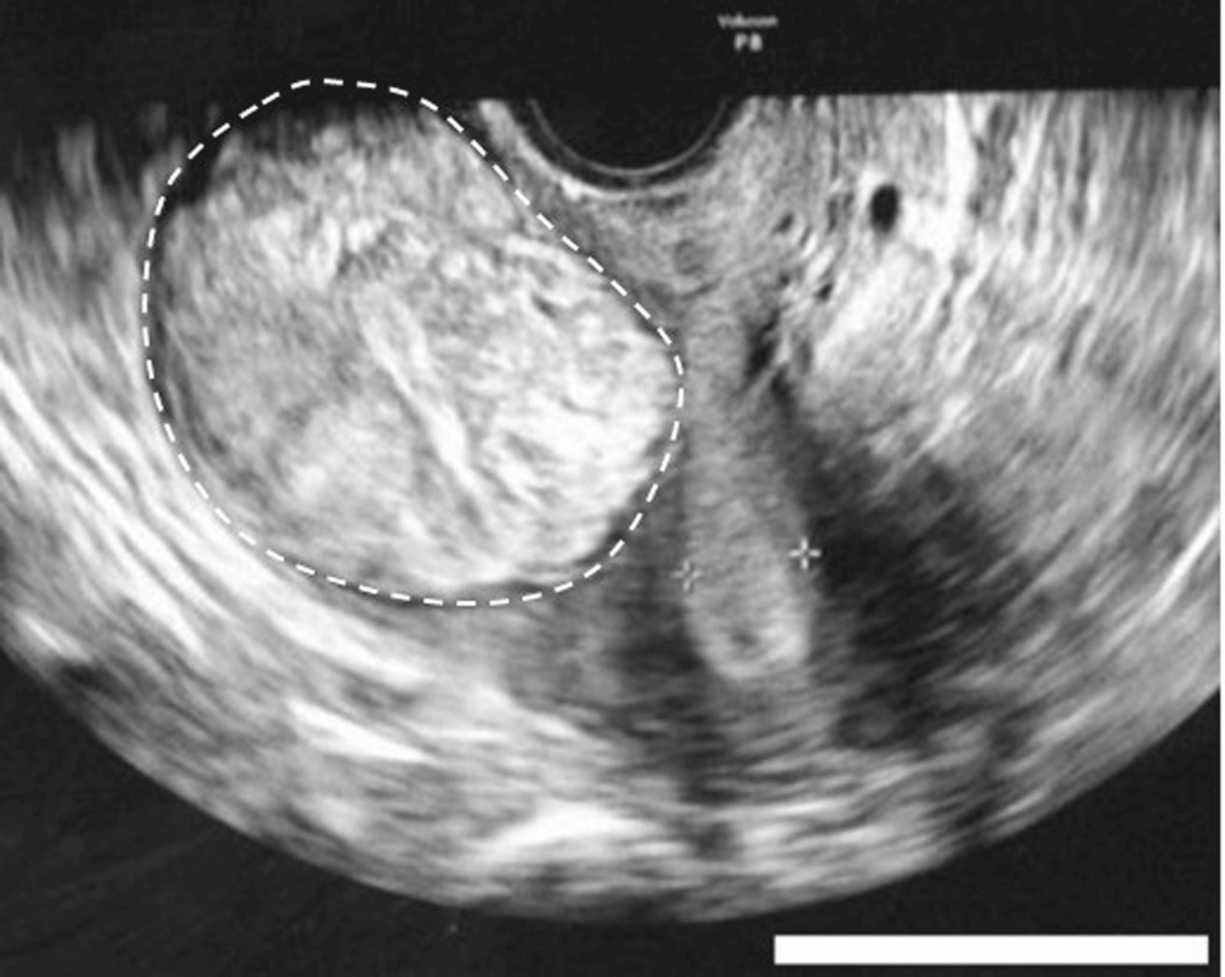
Transvaginal ultrasonography at the initial visit shows a 6 cm uterine myoma in the pouch of Douglas Scale bar is 5 cm.

A 40-year-old woman (gravida 0, para 0) visited our hospital for treatment of infertility. Anthropometric examination revealed a body mass index of 21.4 (weight = 47.8 kg; height = 149.4 cm). She had been diagnosed with a 3 cm uterine myoma at the age of 37 years, but had no history of menstrual abnormalities, including menorrhagia. Upon her first visit to our hospital, the myoma had enlarged to 6 cm in diameter and remained intramural on the posterior wall, without deformation of the uterine cavity, corresponding to Federation of Gynecology and Obstetrics (FIGO) type 5 (Figure 1). During infertility treatment at our facility, the myoma continued to grow, and after 3 cycles of intrauterine insemination, its diameter exceeded 10 cm. Although we recommended myomectomy and advancement of assisted reproductive technology, the patient expressed a strong preference for continued intrauterine insemination, ultimately achieving pregnancy on the fifth attempt.

**Figure 2:**
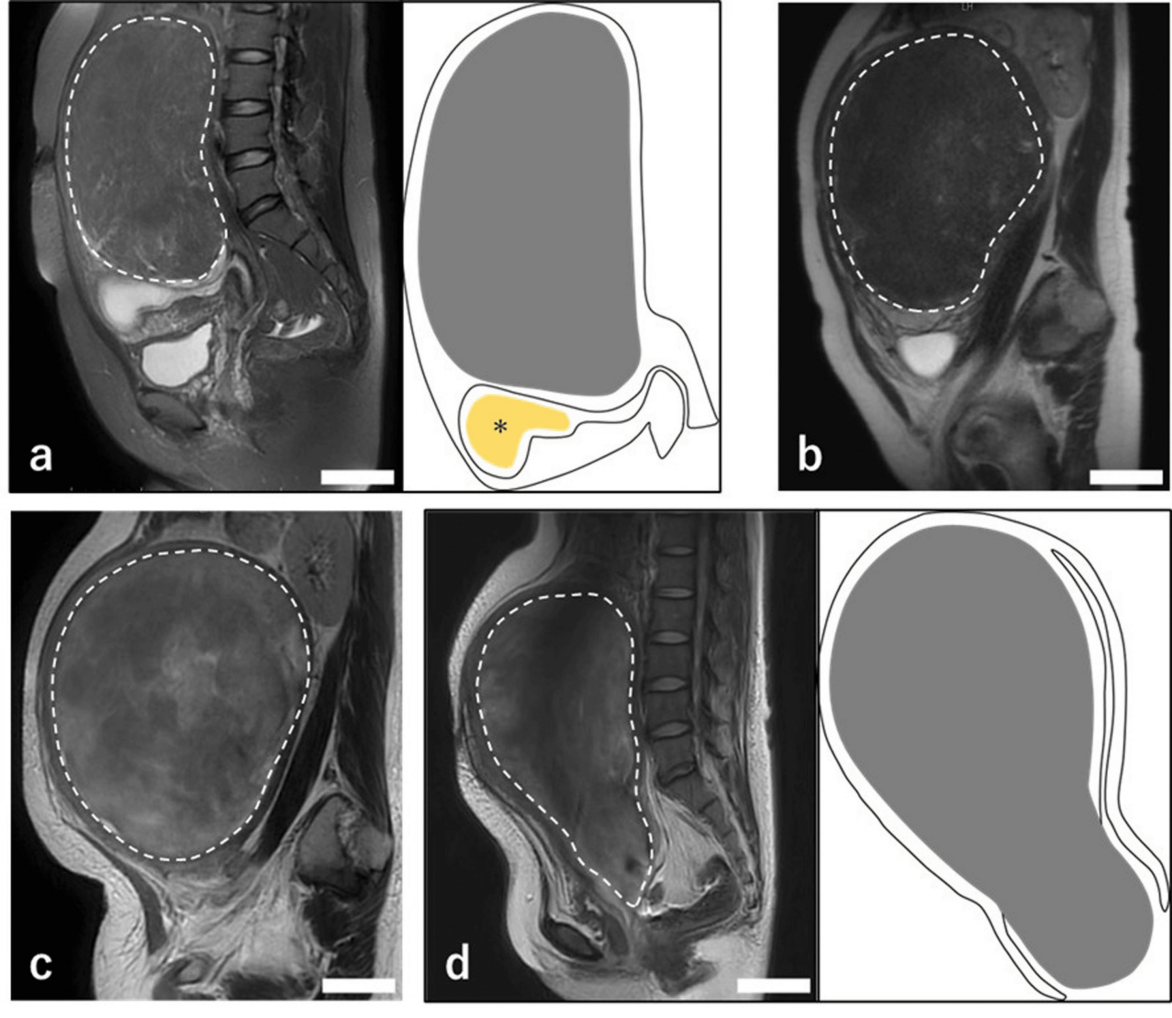
MRI findings of uterine myoma from the initial visit through pregnancy and the postpartum period. Scale bar is 5 cm. (a) (left) Sagittal T2-weighted MRI at 10 weeks of gestation reveals an 18 cm uterine myoma occupying the space from the cephalad aspect of the gestational sac (*) to the epigastric region. (right) Schematic illustration corresponding to the MRI, in which the myoma is highlighted in gray and the gestational sac (*) is indicated in yellow. Image credits: Hitomi Nambu, Author. (b) Sagittal T2-weighted MRI in the second trimester showing that the myoma had grown to 21 cm. (c) Sagittal T2-weighted MRI on postpartum day 49 indicates red degeneration of the uterine myoma. (d) (left) Sagittal T2-weighted MRI and schematic diagram of myoma expulsion on postpartum day 62. The intramural myoma perforates the myometrium and the endometrium. (right) Schematic illustration corresponding to the MRI, in which the myoma is highlighted in gray. Image credits: Hitomi Nambu, Author.

Following conception, the uterine myomas exhibited rapid growth, attaining a diameter of 18 cm by the tenth week of gestation. As the myoma enlarged, it migrated upward from the pouch of Douglas, ultimately occupying a substantial portion of the abdominal cavity and extending from the center to the epigastric region (Figure 2a). Concurrently, extensive deep vein thrombosis (DVT) developed in the lower extremities and is precipitated by the compression of the inferior vena cava by myomas and dehydration resulting from hyperemesis gravidarum. Anticoagulant therapy was initiated with the continuous administration of unfractionated heparin. Unfractionated heparin was initiated at 8,000 IU/day and gradually increased until the target coagulation parameters were achieved, with the final dose reaching 22,500 IU/day. Thereafter, with regular monitoring of blood tests, the dosage was maintained between 20,000 and 24,000 IU/day until delivery. The uterine myomas reached a maximum diameter of 21 cm during the second trimester (Figure 2b), with no significant change in size thereafter until postpartum. The patient delivered vaginally at 38 weeks and 0 days of age. The neonate weighed 2,694 g, with Apgar scores of 8 at 1 min and 10 at 5 min, and umbilical artery blood pH of 7.350. The blood loss during delivery was 2,354 g.

**Figure 3:**
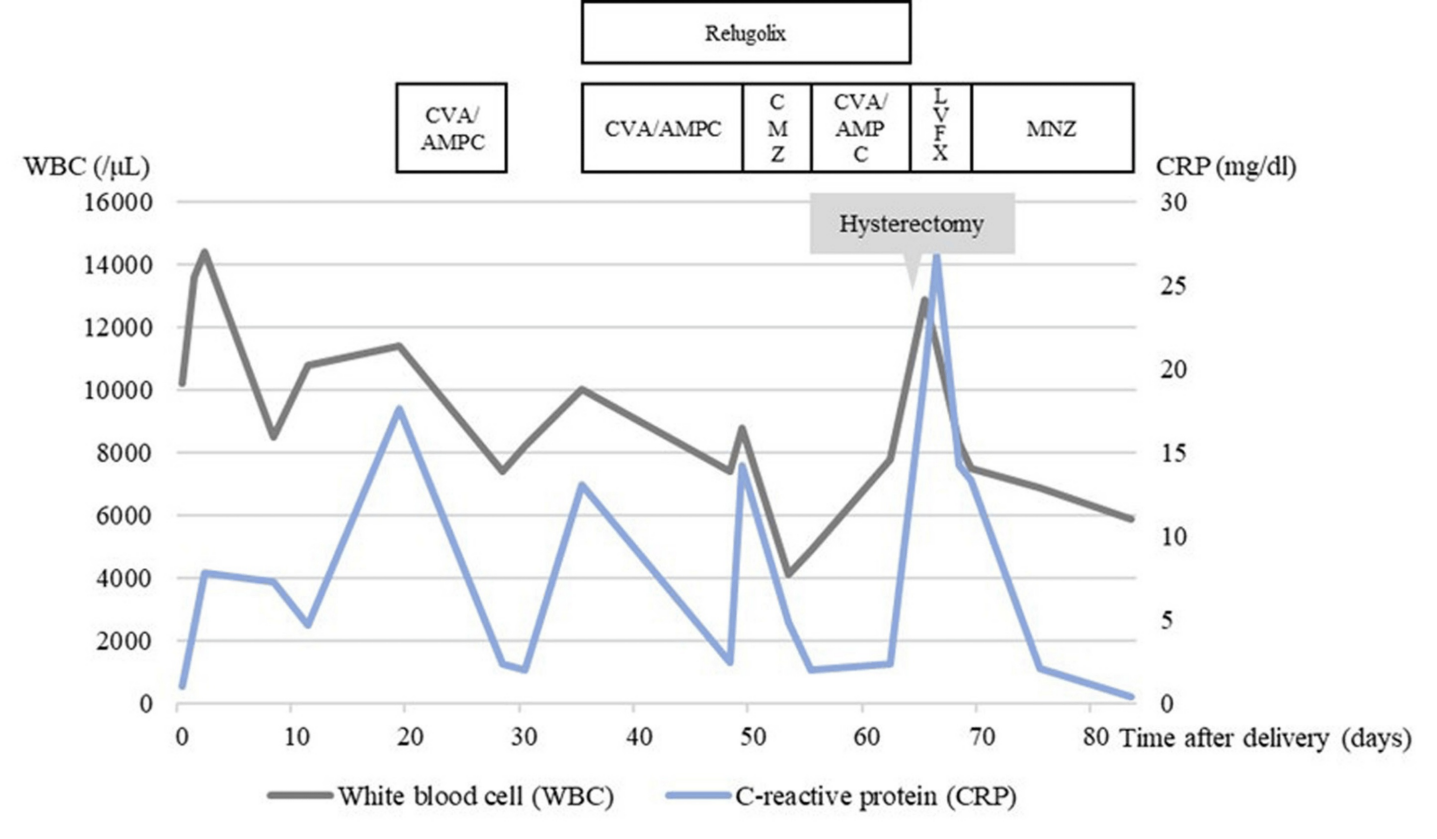
Changes in inflammatory markers during the postpartum period CVA/AMPC, potassium clavulanate/amoxicillin hydrate; CMZ, cefmetazole sodium; LVFX, levofloxacin hydrate; MNZ, metronidazole

For postpartum anticoagulant therapy, the options of breastfeeding with warfarin or weaning with a direct oral anticoagulant (DOAC) were presented, and the patient chose the latter. Oral administration of edoxaban tosilate hydrate 60 mg once daily was initiated. The postpartum hospital course proceeded without complications, including postpartum fever, abdominal pain, or increased bleeding. Ultrasonography was performed immediately after delivery, during hospitalization, and on the day of discharge. Compared with the pre-delivery findings, the uterine myoma showed no change in size, and no findings suggestive of degeneration were observed. The patient was discharged on the eleventh postpartum day, since her hemoglobin level was 10.3 g/dL (reference range: 11.6-14.8 g/dL) and was not regarded as concerning for postpartum anemia. On the twentieth postpartum day, an elevated inflammatory response was detected, although the patient only complained of a small amount of genital bleeding without fever or abdominal pain. Consequently, oral potassium clavulanate/amoxicillin hydrate (CVA/AMPC) was administered. On postpartum day 35, the patient experienced increased genital bleeding without fever, which was interpreted as the resumption of menstruation. Consequently, relugolix, a gonadotropin-releasing hormone (GnRH) antagonist, was initiated with the future option of myomectomy in mind after several months of administration (Figure 3).

On postpartum day 49, the patient presented for fever, abdominal pain, and increased genital bleeding. Pelvic magnetic resonance imaging (MRI) performed on admission revealed a high-signal area at the margin of the intramural myoma on T1-weighted imaging with a slightly hypointense appearance on T2-weighted imaging, suggesting red degeneration of the uterine myoma, a specific type of myoma degeneration characterized by hemorrhagic infarction and necrosis, often resulting from impaired blood circulation (Figure 2c). Although total hysterectomy was considered a definitive treatment option, conservative management was pursued based on the patient's strong desire for a second child.

**Figure 4:**
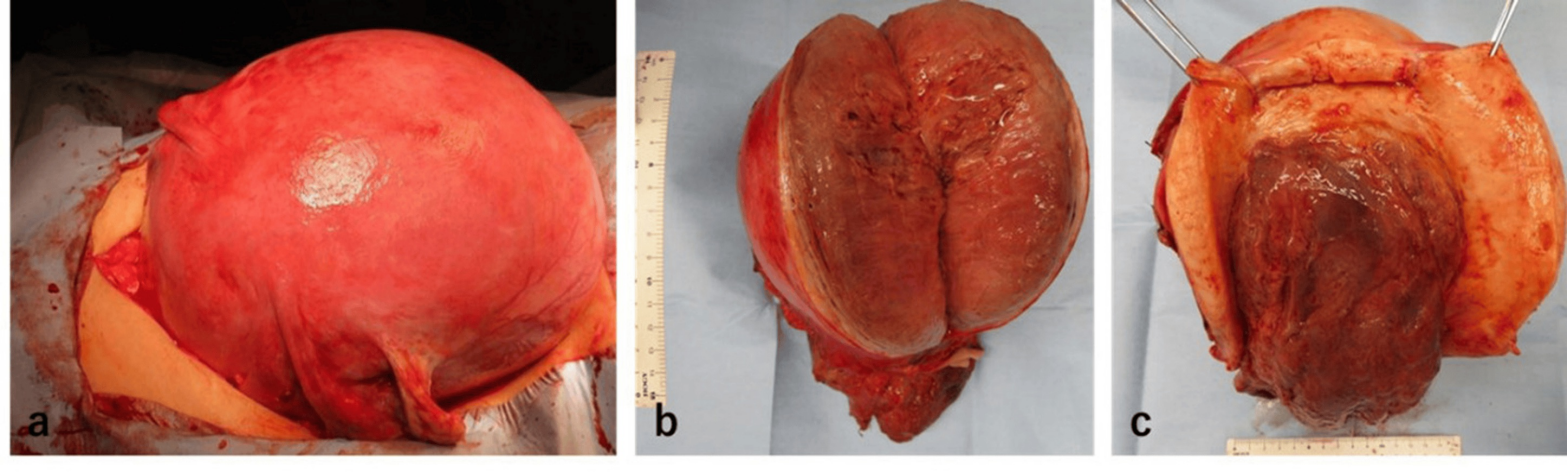
Intraoperative macroscopic findings (a) The uterus was markedly enlarged. (b) The intramural myoma was located predominantly in the posterior wall. (c) A portion of the myoma that had perforated the myometrium and endometrium was found within the uterine cavity, accompanied by a foul odor.

**Figure 5:**
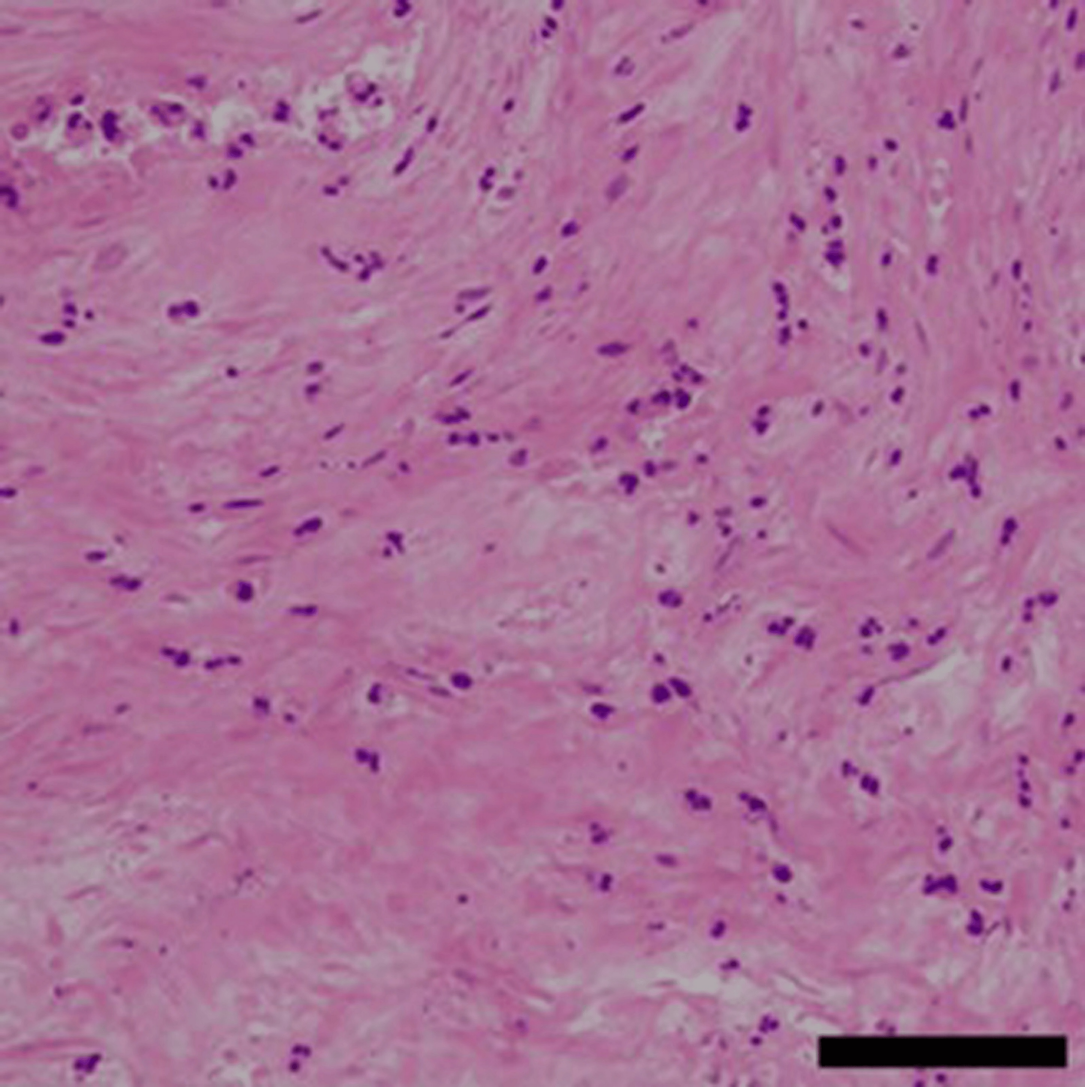
A degenerated leiomyoma exhibiting necrosis and focal neutrophil infiltration was observed. Scale bar is 50 μm.

On postpartum day 62, the patient returned for a third evaluation due to exacerbated abdominal pain. MRI revealed that the intramural myoma had penetrated the endometrium and invaginated into the cervical canal, resulting in a diagnosis of myoma expulsion (Figure 2d). At this juncture, blood tests indicated a mildly elevated inflammatory response, with a white blood cell count of 7,800/μL (reference range: 3,300-8,600/μL), a C-reactive protein level of 2.35 mg/dl (reference range: 0.00-0.14 mg/dL), and a hemoglobin level of 9.8 g/dL. Given the potential risk of progression to sepsis or significant hemorrhage, a simple total abdominal hysterectomy was performed on postpartum day 64. Intraoperatively, the uterus was enlarged to the size of a volleyball (Figure 4a). The primary uterine myoma was located on the posterior wall and penetrated the myometrium and endometrium into the cervical canal, culminating in a myoma expulsion (Figures 4b, 4c). The myoma protruding into the cervical canal was malodorous, and surface cultures revealed *Escherichia coli* and *Morganella morganii*. Pathological examination of the excised uterus revealed extensive necrosis within the myomas and significant neutrophil infiltration, leading to the final diagnosis of pyomyoma (Figure 5).

Intraoperative blood loss was 1,020 g because the hemoglobin level decreased to 6.6 g/dL, and 4 units of concentrated red blood cells were transfused postoperatively. Intravenous infusion of piperacillin/tazobactam (PIPC/TAZ) 4.5 g every 8 hours was initiated postoperatively; however, a drug eruption appeared on postoperative day 1, and the regimen was switched to oral levofloxacin (LVFX) 500 mg once daily. On postoperative day 6, anaerobic bacteria were identified from the culture of the intraperitoneal drain, and the antibiotic was changed to oral metronidazole (MNZ) 250 mg twice daily. The patient's postoperative course was favorable, with improvement in the inflammatory response and anemia, with a hemoglobin level recovering to 9.2 g/dL, and she was discharged on postoperative day 10 (postpartum day 74). Since then, no recurrence of infection has been observed.

## Discussion

In this case, the patient experienced an intra-myoma infection during the puerperium that exhibited a pattern of recurrent exacerbation and amelioration with antibiotic therapy. Ultimately, the intramural myoma breached the myometrium and endometrium, culminating in the expulsion of the myoma. Due to the potential risk of progression to sepsis and significant hemorrhage, a simple abdominal hysterectomy was performed, which resulted in the patient's survival, making this a noteworthy case report. Myoma expulsion during the puerperium is an infrequent occurrence [3], and, to the best of our knowledge, there have been no other documented cases necessitating an abdominal hysterectomy as observed in this case.

The prevalence of pregnancies complicated by uterine myomas has been documented to range from 1% to 10.7% [1], and this incidence is increasing owing to the trend of women marrying and giving birth at an older age. Uterine myomas are known to increase the risk of miscarriage, preterm birth, placenta previa, placental abruption, and postpartum hemorrhage [1]. Myoma expulsion as a postpartum complication is rare [3], and due to its infrequency, there are no established guidelines for its management [4]. In cases of myoma expulsion in non-pregnant women, a study reported transvaginal myomectomy in 46 cases of pedunculated submucosal myomas that prolapsed into the vagina, achieving a success rate of 95.6% [5]. Based on this study, transvaginal myomectomy can be considered as the primary treatment option for non-pregnant patients. Although a similar surgical approach might be a viable treatment option during puerperium, the same study noted that transvaginal resection could not be performed in cases in which access to the stalk was difficult [5]. Therefore, in cases such as the present case, where there is no distinct stalk and the transvaginal approach is challenging, no definitive management strategy has been established.

Uterine myomas that cause deformation of the uterine cavity (FIGO 0-2) have been shown to reduce fertility [6]. It has also been demonstrated that myomectomy improves pregnancy outcomes; therefore, surgical intervention is recommended [6,7]. Conversely, even in cases in which uterine myomas do not deform the uterine cavity, there are indications of reduced pregnancy rates [7]. However, whether myomectomy improves pregnancy rates remains controversial, as reports vary, and currently, there is no consensus [7]. In this particular case, since the myoma was classified as FIGO 5 without deformation of the uterine cavity and the patient wished to continue infertility treatment without undergoing myomectomy, surgical intervention was not performed, and infertility treatment was continued.

A notable aspect of the clinical progression in this case was the enlargement of uterine myomas following the onset of pregnancy. Although it is commonly asserted that most uterine myomas do not undergo significant size changes once pregnancy is established, some myomas have been reported to exhibit a tendency to increase in size [8]. This enlargement is particularly pronounced during the first trimester, especially up to approximately the tenth week of gestation [8]. According to a study by Lee HJ and colleagues, the average rate of volume increase was 12 ± 6%, with a maximum increase of 25% [8]. In the second trimester, the growth pattern varies based on the myoma size; myomas smaller than 6 cm tend to grow, whereas those 6 cm or larger either remain unchanged or decrease in size. In the third trimester, myomas frequently tend to diminish in size [9,10]. In this particular case, the myoma measured approximately 10 cm prior to pregnancy and rapidly expanded to 18 cm by approximately the tenth week of gestation. It then continued to increase slowly, reaching a maximum diameter of 21 cm in the second trimester, after which it remained largely unchanged. Rapid enlargement of the uterine myoma also emerged as a significant concern in the management of pregnancy and delivery in this case.

We conducted a review of the documented cases of postpartum myoma expulsion since 2000, identifying a total of 13 cases, including our patient (Table 1) [2-4,11-19]. The myomas were located not only in the submucosal regions, which are more predisposed to expulsion, but also included seven cases of intramural myomas and two cases of cervical myomas. Similar to the current case, these observations imply that intramural myomas may undergo expulsion under the influence of infection or degeneration. Furthermore, ten cases involved myomas that exceeded 10 cm in size, suggesting that the size of the myoma may be a contributing factor. The onset of expulsion varied significantly, occurring immediately after delivery to as late as seven weeks postpartum. Notably, infection was present in nine of the 13 cases, strongly indicating its role in the initiation of myoma expulsion. In most cases, the myomas are pedunculated, and transvaginal or transabdominal myomectomy is the preferred treatment. Only one case, as reported by Pieh-Holder et al., involved a total hysterectomy, specifically a vaginal hysterectomy, due to complications from irreducible uterine inversion [15]. To our knowledge, this report represents the first case of a patient requiring a GnRH antagonist due to the cessation of lactation and persistent menstrual bleeding, who subsequently underwent a total abdominal hysterectomy. Pathological examination revealed myoma degeneration in all cases, which was believed to result from reduced blood flow to the myoma during the postpartum period.

**Table 1:** Summary of documented cases of myoma expulsion during puerperium since 2000

Author (year)	Age (years old)	Complaints	Location	Size (cm)	Delivery mode	Time to onset after delivery	Presence of pyomyoma	Intervention	Pathology
Mason TC (2002) [11]	29	Urinary retention, vaginal bleeding, abdominal pain	N/A	15×15×3	Vaginal delivery	4 weeks	-	Vaginal myomectomy	Degenerating leiomyoma
Thorpe-Beeston JG, et al. (2002) [12]	31	Vaginal bleeding, abdominal pain	Submucosal myoma	8×7×6	Vaginal delivery	Third labor	-	Spontaneously delivery	Degenerating leiomyoma
Murakami T, et al. (2007) [13]	32	Fever, vaginal bleeding	Submucosal myoma	N/A	Cesarean section	16 days	+	Abdominal myomectomy	Degenerating leiomyoma
Torrance SM, et al. (2009) [14]	28	Fever, abdominal pain, smelly discharge, a prolapsed vaginal mass	Intramural myoma	11×6×6	Vaginal delivery	18 days	+	Abdominal myomectomy	Degenerating leiomyoma
Pieh-Holder KL, et al. (2014) [15]	40	Vaginal bleeding	Intramural myoma	5	Vaginal delivery	7 weeks	-	Vaginal hysterectomy	Leiomyoma with severe ischemic changes
DeMaio A, et al. (2015) [16]	N/A	Fever, a prolapsed vaginal mass	Intramural myoma	27×10	Vaginal delivery	5 weeks	+	Vaginal myomectomy	Degenerating leiomyoma
Sagoo B, et al. (2015) [17]	36	Fever, abdominal pain, smelly discharge	Intramural myoma	12×11×9	Cesarean section	6 weeks	+	Vaginal myomectomy	N/A
Elgonaid W, et al. (2017) [4]	35	Abdominal pain, smelly discharge	Intramural myoma	18×16×8	Vaginal delivery	35 days	N/A	Vaginal myomectomy	Infarcted leiomyoma
Zhang J, et al. (2018) [18]	22	Fever, a prolapsed vaginal mass, smelly discharge	Submucosal myoma	38×6×2	Vaginal delivery	12 days	+	Vaginal myomectomy	Degenerating leiomyoma with infarction
Nkwabong E. (2018) [19]	25	Fever, abdominal pain, smelly discharge, urinary retention	Intramural myoma	10	Vaginal delivery	5 weeks	+	Vaginal myomectomy	Degenerating leiomyoma
Wang J, et al. (2021) [2]	24	Fever, abdominal pain	Submucosal myoma	17×10×9	Vaginal delivery	40 days	+	Vaginal and laparoscopic myomectomy	Degenerating leiomyoma with significant suppurative necrosis
Li L, et al. (2023) [3]	24	Fever, a prolapsed vaginal mass	Submucosal myoma	18×10×4	Cesarean section	1 day	+	Vaginal myomectomy	Leiomyoma with coagulation necrosis
The current case (2025)	40	Fever, abdominal pain	Intramural myoma	21	Vaginal delivery	62 days	+	GnRH antagonist + Abdominal hysterectomy	Degenerating leiomyoma with infection and necrosis

The precise mechanisms underlying the onset and risk factors associated with the expulsion of uterine myomas remain poorly understood. Previous studies have documented cases following uterine artery embolization, a GnRH agonist treatment, and during the postpartum period, indicating that these may represent potential risk factors [3]. A commonality among these scenarios is the reduction in blood flow to the myoma, which leads to necrosis or degeneration of the tumor. Notably, the postpartum period creates conditions conducive to the expulsion of uterine myomas because of the strong uterine contractions associated with uterine involution and anatomical changes, such as cervical dilation; thus, myoma expulsion is believed to be facilitated during this time [18]. In the present case, two overlapping risk factors were identified: physiological changes in the postpartum period and a history of treatment with a GnRH antagonist.

Notably, the uterine myoma in this case was initially located within the myometrium. Although there are limited reports of intramural myomas leading to myoma expulsion, it has been proposed that, in the presence of an infection, an intramural myoma may assume a submucosal-like form, facilitating myoma expulsion. Potential infection pathways include direct infection from the uterine cavity, spread from adjacent organs, and hematogenous or lymphatic transmission [11]. It is hypothesized that following postpartum uterine involution, the blood supply to the large myoma is obstructed, resulting in degeneration and necrosis. Moreover, a GnRH antagonist can exacerbate impaired circulation, leading to a rapid and potent reduction in uterine blood flow in the puerperal uterus and subsequent ischemic changes in the myoma [20]. These ischemic changes are thought to have led to a secondary bacterial infection of the degenerated and necrotic myoma. The infection compromised the integrity of the surrounding myometrium and endometrium. With additional pressure from uterine contractions and the myoma's weight, the myoma ultimately penetrated the myometrium and endometrium, leading to myoma expulsion.

In the present case, as in previously documented cases, a substantial intramural myoma penetrated the uterine wall and endometrium due to degeneration and infection during the postpartum period, subsequently transitioning to a submucosal myoma-like form. However, given that the myoma nearly entirely supplanted the anterior wall of the uterine body and exhibited minimal protrusion into the cervical canal, myomectomy was considered challenging, leading to the decision to perform abdominal total hysterectomy. By excising the uterus, which served as the infectious focus, the progression to sepsis was averted, resulting in a favorable outcome. The unique hormonal milieu of pregnancy and the puerperium can cause hormonal agents to exhibit atypical or unexpected responses, thus warranting careful consideration and caution.

## Conclusions

We present the first documented case of myoma expulsion during the puerperal period, precipitated by infection, which posed a risk of progression to sepsis but was successfully managed through abdominal hysterectomy. Typically, myoma expulsion is associated with stalks in submucosal myomas for which vaginal myomectomy is the standard intervention. However, in cases where the myoma is extensively and deeply embedded within the myometrium without a stalk, myomectomy presents significant challenges. Accurate assessment of the origin of the myomas and the degree of protrusion is essential when formulating a treatment strategy for myoma expulsion. In this case, a total hysterectomy was the only option available as a radical treatment, but if the myomectomy had been performed during infertility treatment or immediately after giving birth, the hysterectomy might have been avoided.
